# Partial Improvement of Spatial Memory Damages by Bone Marrow Mesenchymal Stem Cells Transplantation Following Trimethyltin Chloride Administration in the Rat CA1

**DOI:** 10.32598/BCN.9.10.90

**Published:** 2019-11-01

**Authors:** Soheila Madadi, Majid Katebi, Mina Eftekharzadeh, Ahmad Mehdipour, Bagher Pourheydar, Mehdi Mehdizadeh

**Affiliations:** 1. Department of Anatomy, Faculty of Medicine, Arak University of Medical Sciences, Arak, Iran.; 2. Department of Anatomy, Faculty of Medicine, Hormozgan University of Medical Sciences, Bandar Abbas, Iran.; 3. Department of Anatomy, Faculty of Medicine, Iran University of Medical Sciences, Tehran, Iran.; 4. Department of Tissue Engineering, Faculty of Advanced Medical Sciences, Tabriz University of Medical Sciences, Tabriz, Iran.; 5. Neurophysiology Research Center, Department of Anatomy, Faculty of Medicine, Urmia University of Medical Sciences, Urmia, Iran.; 6. Cellular and Molecular Research Center, Department of Anatomy, Faculty of Medicine, Iran University of Medical Sciences, Tehran, Iran.

**Keywords:** Trimethyltin Chloride (TMT), Mesenchymal Stem Cells (MSCs), Hippocampus, Spatial Memory

## Abstract

**Introduction:**

Trimethyltin Chloride (TMT) is a neurotoxin that can kill neurons in the nervous system and activate astrocytes. This neurotoxin mainly damages the hippocampal neurons. After TMT injection, behavioral changes such as aggression and hyperactivity have been reported in animals along with impaired spatial and learning memory. Hence, TMT is a suitable tool for an experimental model of neurodegeneration. The present study aims to determine the palliative effects of Bone Marrow-derived Mesenchymal Stem Cells (BM-MSCs) on the hippocampi of rats damaged from TMT exposure.

**Methods:**

We assigned 28 male Wistar rats to the following groups: control, model, vehicle, and treatment. The groups received Intraperitoneal (IP) injections of 8 mg/kg TMT. After one week, stem cells were stereotactically injected into the CA1 of the right rats’ hippocampi. Spatial memory was determined by the Morris Water Maze (MWM) test 6 weeks after cell transplantation. Finally, the rats’ brains were perfused and stained by cresyl violet to determine the numbers of cells in the Cornus Ammonis (CA1) section of the hippocampus. We assessed the expressions of Glial Fibrillary Acidic Protein (GFAP) and Neuronal-specific Nuclear (NeuN) proteins in the right hippocampus by Western blot.

**Results:**

The MWM test showed that the treatment group had significantly higher traveled distances in the target quarter compared with the model and vehicle groups (P<0.05). Based on the result of cell count (Nissl staining), the number of cells increased in the treatment group compared with the model and vehicle groups (P<0.05). Western blot results showed up-regulation of GFAP and NeuN proteins in the model, vehicle, and treatment groups compared with the control group.

**Conclusion:**

Injection of BM-MSCs may lead to a behavioral and histological improvement in TMT-induced neurotoxicity by increasing the number of pyramidal neurons and improving memory.

## Highlights

- The transplantation of Bone Marrow-derived Mesenchymal Stem Cells (BM-MSCs) increased the number of pyramidal neurons in the damaged hippocampus.
- The BM-MSCs transplantation alleviated impaired memory caused by trimethyltin chloride exposure.
- The transplantation of BM-MSCs increased neuronal specific nuclear protein expression and decreased the expression of the glial fibrillary acidic protein.

## Plain Language Summary

The hippocampus is a key area in the cortex of the brain. It is associated with memory and learning and has a vital role in the formation of new memory, spatial analysis, as well as integration and transfer of information from short-term to long-term memory. Despite the vital role of the hippocampus in memory and spatial learning, this organ is unprotected and very sensitive and vulnerable to injuries. The hippocampus gets injured by hypoxia, encephalitis, infection, Alzheimer disease, stroke, ischemia, and especially brain trauma. In the case of brain infections, in the limbic, amygdala, and hippocampal systems, the behavioral changes are observed due to short-term memory and spatial recognition impairment. Studies have shown that the mammalian hippocampus has neurogenesis ability throughout life. However, it cannot overcome hippocampus damages. Considering the high sensitivity of the hippocampal tissue and its essential role in memory and learning, it is very important to find a way to reduce its damage and treat it after injuries. Trimethyltin chloride (TMT) is a neurotoxin that can kill neurons in the nervous system. This neurotoxin mainly damages the hippocampal neurons. Hence, TMT is a suitable tool for an experimental model of neurodegeneration. Today, stem cells are a suitable treatment method for the improvement of nervous system disease. So that following transplantation of stem cells, neuron regeneration occurs in damaged regions. The present study showed that using bone marrow mesenchymal stem cells decreases hippocampal lesions by increasing the number of pyramidal neurons, improving behavioral performance and memory, and reducing cognitive deficits.

## 1. Introduction

The central nervous system is the target of environmental toxins (Liu et al., 2006). For instance, the hippocampus, especially the Cornus Ammonis (CA) area which plays a crucial role in memory and spatial learning, is susceptible to toxins (Annane, 2009). Trimethyltin chloride (TMT) is a potent neurotoxin that causes severe neuronal death, particularly in the hippocampus. Areas most affected include CA1, CA3 and hilus (Geloso, Vinesi, & Michetti, 1996, 1997). The neurological effects of TMT were reported in 1955 for the first time. TMT activates glial cells, including astrocytes, both in vivo and in vitro conditions (Haga, Haga, Aizawa, & Ikeda, 2002; Röhl & Sievers, 2005). Following the TMT insult, there is a transient increase in the Glial Fibrillary Acidic Protein (GFAP) cell marker (Brock & O’callaghan, 1987).

Symptoms such as aggression, tremor, convulsions, hyperactivity, and cognitive impairment have been reported following exposure to TMT (Bushnell, 1990; Earley, Burke, & Leonard, 1992; Moser, 1996; Woodruff, Baisden, Cannon, Kalbfleisch, & Freeman, 1994). Therefore, the administration of TMT provides a suitable model for neuronal death and behavioral problems associated with cognitive functions (Brabeck et al., 2002; Geloso et al., 2004). Neural Stem Cells (NSCs) that reside in the subgranular zone of the hippocampal dentate gyrus (Cameron, McEwen, & Gould, 1995) and the subventricular region of the lateral wall of the lateral ventricle can undergo neurogenesis in the adult brain (Gage, 2000). Neurogenesis is stimulated in response to brain injury (Emery et al., 2005) and survival of newborn cells by endogenous NSCs effectively repair neuronal damage (Arvidsson, Collin, Kirik, Kokaia, & Lindvall, 2002). However, neurogenesis cannot completely treat neuronal damage (Munoz, Stoutenger, Robinson, Spees, & Prockop, 2005).

The two features of undifferentiated stem cells are the ability for self-renewal and differentiation into mature cells (Wagers & Weissman, 2004). Embryonic and adult stem cells are the two main types of stem cells. Adult stem cells are present in different organs of the body. These cells are easily accessible and have no ethical limitations for their use (Mauron & Jaconi, 2007; Morsczeck et al., 2008). Mesenchymal Stem Cells (MSCs) are isolated from Bone Marrow (BM) stroma. These multipotent cells can differentiate into different cell types (Azizi, Stokes, Augelli, DiGirolamo, & Prockop, 1998; Pittenger et al., 1999), including mesenchymal cell types such as bone, cartilage, fat, and muscle as well as ectodermal lineage (neurons, astrocytes, and oligodendrocytes) (Minguell, Erices, & Conget, 2001; Sanchez-Ramos, 2002). Recently, studies report the therapeutic potential of BM-MSCs in various pathological conditions (Zhang et al., 2005). The present study aims to determine the treatment effects of BM-MSCs in a rat model of the hippocampus damaged by exposure to TMT.

## 2. Methods

### 2.1. Experimental groups and drugs

In this study, we used 28 male Wistar rats weighing 250–300 g. The rats were kept in a temperature-controlled room (20ºC–22ºC) on a 12:12-h light:dark cycle. Water and standard pellet food were provided for them ad libitum. We randomly divided the rats into four groups (7 rats in each group): 1. No TMT injection (control); 2. A single intraperitoneal (IP) injection of 8 mg/kg body weight of TMT in a volume of 1 mL/kg body weight dissolved in normal saline (model) (Corvino et al., 2005); 3. A single injection of 8 mg/kg body weight TMT, followed by 4 μL of phosphate-buffered saline (PBS) stereotactically injected into the CA1 area of the right hippocampus one week later (vehicle); and 4. A single injection of 8 mg/kg body weight TMT, followed by 4 μL cell suspension stereotactically injected into the CA1 area of the right hippocampus 1 week later (treatment) (Munoz et al., 2005).

### 2.2. Cell culture

We isolated BM-MSCs, by separating the tibia and femur bones from two 8-week-old male Wistar rats, as previously reported (Hu et al., 2011). Briefly, we used a syringe with a 20-gauge needle to flush the BM-MSCs from the bones of donor adult rats. The resultant cells were cultured in Dulbecco’s modified Eagle’s medium (DMEM; Gibco) that contained 10% fetal bovine serum (FBS; Gibco), 100 U/mL penicillin and 100 μg/mL streptomycin (Gibco). The cells were allowed to incubate at 37ºC in a humidified chamber filled with 95% air and 5% CO _2_ . After 48 h, the medium that contained non-adherent cells was removed from the flask by washing with PBS and replaced by fresh medium. The medium was changed every 3 days. When the BMSCs were 80% confluent, we passaged them at a 1:2 ratio by incubation with 0.25% trypsin/EDTA (Gibco) for 3 min. The BM-MSCs were labeled with CellTracker™ chloromethylbenzamido Dil (CM-Dil, red fluorescence; Invitrogen) before injection to facilitate cell tracking after transplantation. Briefly, the cells were incubated with CM-Dil for 5 min at 37ºC and 15 min at 4ºC. We used cells of the third or fourth passages for the experiments.

### 2.3. Stereotaxic surgery

The rats were anesthetized with IP injections of ketamine (100 mg/kg) and xylazine (10 mg/kg), then mounted in a stereotaxic apparatus (Stoelting, Wood Dale, IL, USA). Then we drilled their skulls at a suitable location for the insertion of a Hamilton microsyringe (Hamilton, Reno, NV, USA). The location for the CA1 of the right hippocampus was selected based on the Paxinos and Watson rat brain atlas (A/P: −3.8 mm; M/L: +2.2 mm; D/V: 2.9 mm) (Paxinos, Watson, Pennisi, & Topple, 1985). Approximately 4 μL of the cell suspension (100000 BM-MSCs in 4 μL PBS) was slowly injected into the zone at 0.5 μL/min unilaterally. We evaluated cell viability with trypan blue staining before the injection. Nonviable cells were calculated with a Neubauer slide. The needle remained in place for 2 min after the injection, and then it was slowly withdrawn. The vehicle group received an equal volume of PBS into the CA1 of the right hippocampus. The skin of the scalp was sutured. Finally, the rats were allowed to recover in a warm location and then were returned to the cages.

### 2.4. Morris Water Maze (MWM)

Six weeks after the cell injection (7 weeks after TMT injection), we assessed the spatial memory of the rats by the Morris Water Maze (MWM) test. The MWM test comprised a circular tank with a diameter of 170 cm and a depth of 90 cm, filled with water to a height of 25 cm. The water temperature was 22±1ºC (Asl et al., 2013). The tank was composed of four quadrants, and a hidden platform (18 cm diameter) placed 2 cm underwater in the center of one of the quadrants. The animals received training for four consecutive days (training days). Each day consisted of 4 trials. If an animal could not find the platform in 60 s, we would place the animal on the platform for 20 s. We used a video camera (Nikon, Melville, NY, USA) linked to a computer located above the tank to record the time taken to reach the hidden platform (escape latency) and distance spent to achieve the hidden platform (traveled distance). After four days of training, on the fifth day, each rat was given 60 s for the probe trial (probe day). There was no platform in the probe trial.

### 2.5. Perfusion, cresyl violet staining, and neuron counts (Nissl staining)

After the MWM test, three rats of each group were anesthetized by IP injections of ketamine (100 mg/kg) and xylazine (10 mg/kg). Next, they were perfused in the transcardiac area with 150–200 mL of normal saline solution, followed by 250–300 mL of 4% paraformaldehyde in 0.1 M phosphate buffer (pH: 7.4). We removed the animals’ brains, embedded them in paraffin, and cut the tissues into 5-μm coronal sections. For Nissl staining, the sections were deparaffinized and rehydrated and then stained with 0.1% cresyl violet. For each rat, we assessed the number of CA1 pyramidal neurons by counting 3 sections of the right hippocampus at 120 μm intervals at 400x magnification with a light microscope and blinded investigation. Only cells were counted that had clear nuclei and nucleoli. Images were taken by a digital camera (Olympus, DP 11, Japan) connected to a microscope (Olympus Provis, Ax70, Japan) at 400x magnification.

### 2.6. Western blot

Western blot was used to quantify the expression of GFAP and Neuronal-specific Nuclear (NeuN) in the rats. After the MWM, three rats of each group were sacrificed by injections of ketamine (100 mg/kg) and xylazine (10 mg/kg). The right hippocampus of each rat was removed, frozen in liquid nitrogen, and stored at −80ºC. The samples were homogenized with ice-cold lysis buffer that contained a mixture of RIPA buffer with a protease inhibitor cocktail (1:10) for 1 h, then centrifuged (Eppendorf, Hamburg, Germany) at 13000 g for 20 min at 4ºC. We removed the supernatant from the samples. Then, the protein concentration was determined with a Bio-Rad protein assay buffer (Bio-Rad, Hercules, CA, USA), where 100 μg protein aliquots from each sample was denatured with sample buffer that consisted of 6.205 mM Tris-HCl, 2% Sodium Dodecyl Sulfate (SDS), 50 mM 2-mercaptoethanol (2-ME), 0.01% bromophenol blue, and 10% glycerol at a temperature of 99ºC for 5 min. Next, the protein extract was separated on 10% SDS polyacrylamide gels for 90 min at a voltage 120 V, and then it transferred to nitrocellulose membranes.

Non-specific binding sites were blocked with 5% skim milk powder that was soluble in TTBS buffer that consisted of Tris (50 Mm), 0.05% Tween20, and 1.5% NaCl at pH: 7.5 for 90 min at room temperature. The membranes were incubated overnight with monoclonal antibody to NeuN (1:1000; Millipore, USA) and a polyclonal antibody to GFAP (1:50000; Abcam, USA) at 4ºC. Then, blots were exposed to HRP-conjugated secondary antibody (1:10000; Sigma Aldrich, USA) for 1 h at room temperature. β-Actin antibody (1:1000; Sigma Aldrich, USA) was used to recognize the endogenous standard for normalization. The bands were observed after the addition of BCIP solution, then scanned and analyzed by Uvitec software V. 12.6.

### 2.7. Statistical analysis

SPSS 16 was used to analyze the results (Mean±SEM) of this study. The significant difference was assessed with one-way ANOVA with the Tukey test for multiple comparisons. P≤0.05 were considered statistically significant.

## 3. Results

### 3.1. Effect of cell graft on spatial memory in the MWM test

**Figure 1.**
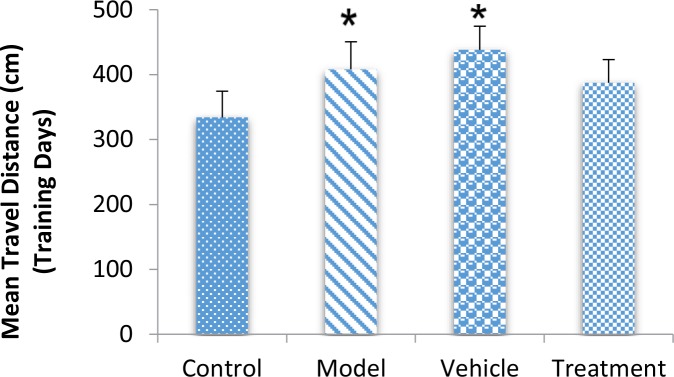
Effect of BM-MSC transplantation on a TMT-induced memory deficit model during training days for the Morris Water Maze (MWM) test TMT increased the traveled distance to reach the hidden platform ( ^*^ P<0.05; model and vehicle groups vs. control group). Grafted BM-MSCs decreased the traveled distance to reach the hidden platform (P>0.05).

**Figure 2.**
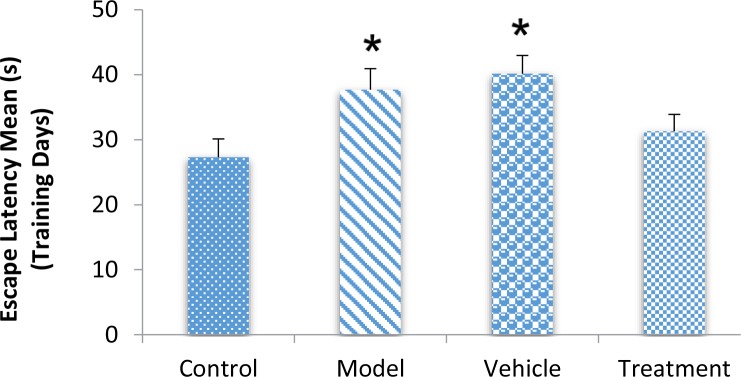
TMT increased escape latency to reach the hidden platform ^*^ P<0.05; model and vehicle groups vs. control group. Grafted BM-MSCs decreased escape latency to reach the hidden platform (P>0.05). Model and vehicle groups vs. control group). Grafted BM-MSCs decreased escape latency to reach the hidden platform (P>0.05). The presence of bone marrow-derived mesenchymal stem cells at the damage site

According to the MWM test results, the BM-MSC graft alleviated impaired memory by TMT exposure. During training days, traveled distance increased significantly in the model (407.98±42.60 cm) and vehicle (438.18±36.39 cm) groups compared with the control (334.01±40.41 cm) group, escape latency also significantly increased in the model (37.68±3.24 s) and vehicle (40.14±2.81 s) groups compared with the control (27.29±2.82 s) group (P<0.05) (Figure 1 and Figure 2). These findings indicated a defect in learning performance following TMT toxicity. The traveled distance decreased in the treatment (387.35±35.75 cm) group compared with the model and vehicle groups. The escape latency also decreased in the treatment (31.30±2.58 s) group compared with the model and vehicle groups. There were no significant differences between these groups for traveled distance and escape latency (P>0.05) (Figure 1 and Figure 2).

**Figure 3.**
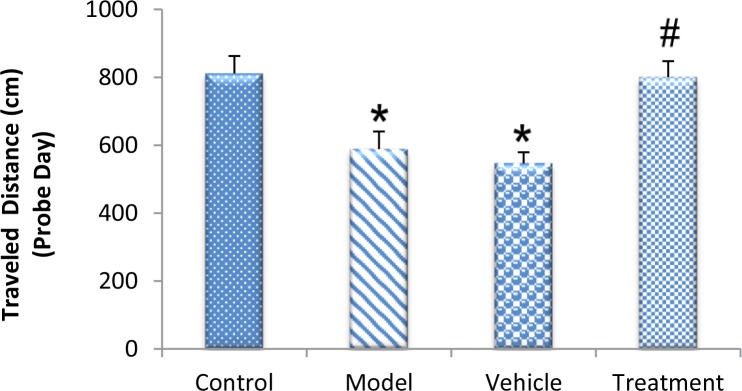
Effect of BM-MSC transplantation on a TMT-induced memory deficit model on the probe day of the Morris Water Maze (MWM) test TMT decreased the traveled distance in the target quarter ( ^*^ P<0.05; model and vehicle groups vs. control group). Grafted BM-MSCs increased the traveled distance in the target quarter ( ^#^ P<0.05; treatment group vs. model and vehicle groups).

**Figure 4.**
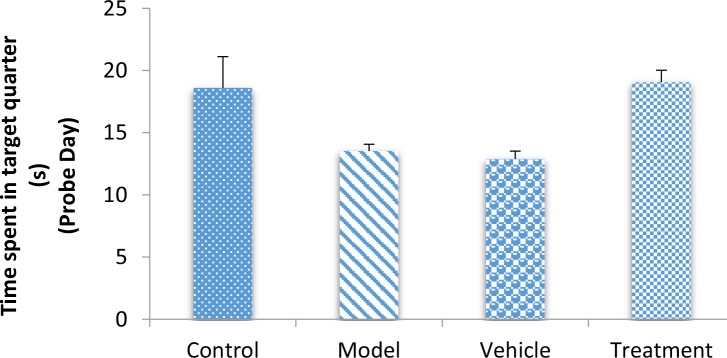
TMT decreased the time spent in the target quarter ( ^*^ P<0.05; model and vehicle groups vs. control group). Grafted BM-MSCs increased the time spent in the target quarter ( ^#^ P<0.05; treatment group vs. model and vehicle groups).

On the fifth (probe) day, the treatment (799.80±47.59 cm) group traveled a significantly higher distance in the target quarter that contained the platform during the training days compared with the vehicle (546.48±32.68 cm) and model (588.51±51.75 cm) groups (P<0.05) (Figure 3). The vehicle and model groups had a significant decrease in the traveled distance in the target quarter compared with the control (811.32±51.32 cm) group (P<0.05) (Figure 3). The time spent in the target quarter decreased significantly in the model (13.52±0.55 s) and vehicle (12.88±0.63 s) groups compared with the control (18.59±2.51 s) group (P<0.05) (Figure 4), whereas it increased in the treatment (19.05±0.96 s) group compared with the model and vehicle groups. However, these findings did not significantly differ between the groups (P>0.05) (Figure 4).

**Figure 5.**
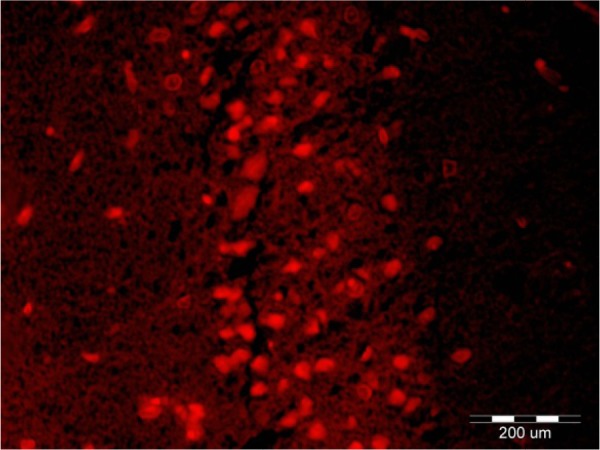
Fluorescence image of the coronal section of the Cornus Ammonis (CA1) hippocampus Demonstrates the distribution of CellTracker™ chloromethylbenzamido-Dil (CM-Dil)-labeled BM-MSCs. Magnification: 400×.

Intracerebral distribution of the CM-Dil-labeled BM-MSCs after transplantation was assessed with fluorescent microscopy (Figure 5). Effect of cell graft on neuronal density in the Cornus Ammonis (CA1) hippocampus. In the present study, we counted the number of normal cells in the pyramidal layer of the CA1 hippocampus.

**Figure 6.**
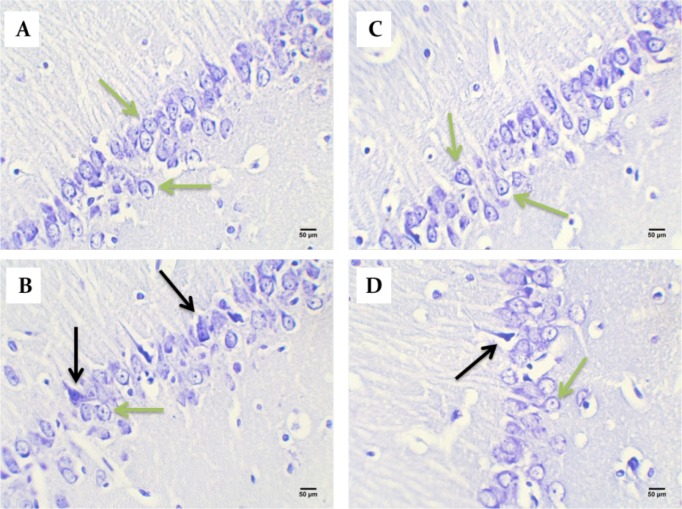
Photomicrographs of the coronal sections of the Cornus Ammonis (CA1) region of the hippocampus with Nissl staining A. Control; B. Model; C. Treatment; and D. Vehicle groups Magnification: 400×. Green arrows show intact neurons, and black arrows represent necrotic (nonviable) neurons.

**Figure 7.**
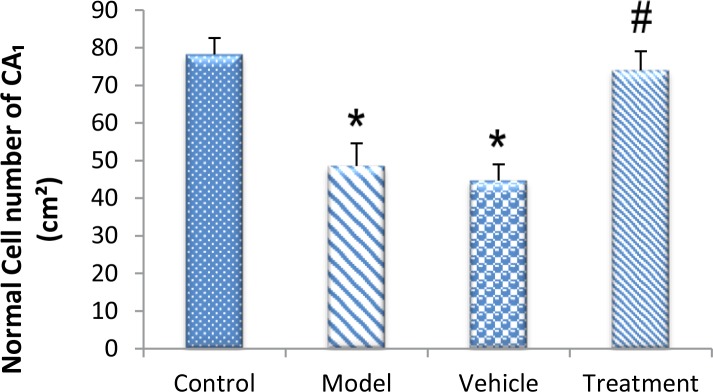
Protective effect of BM-MSC transplantation on TMT-induced cell death in the Cornus Ammonis (CA1) region of the hippocampus TMT treatment decreased the number of normal cells ( ^*^ P<0.05; model and vehicle vs. control groups), and the BM-MSCs graft increased the number of normal cells ( ^#^ P<0.05; treatment group vs. model and vehicle groups).

Based on the cell counts (Nissl staining), TMT led to a significant decrease (and degeneration) of neurons in the CA1 hippocampus in the vehicle (44.67±4.32) and model (48.56±6.03) groups compared with the control (78.22±4.37) group (P<0.05) (Figure 6 and Figure 7). BM-MSCs graft increased the number of normal cells in the treatment (74±5.06) group compared with the vehicle and model groups (P<0.05) (Figure 6 and Figure 7).

### 3.2. Effect of cell graft on glial fibrillary acidic protein and neuronal-specific nuclear expressions

**Figure 8.**
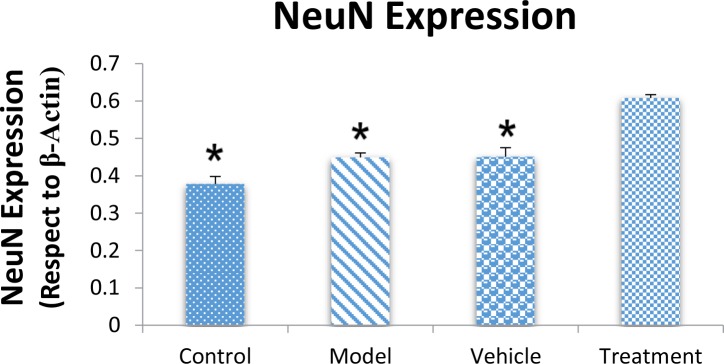
Western blot analysis of neuronal-specific nuclear (NeuN) protein expressions

**Figure 9.**
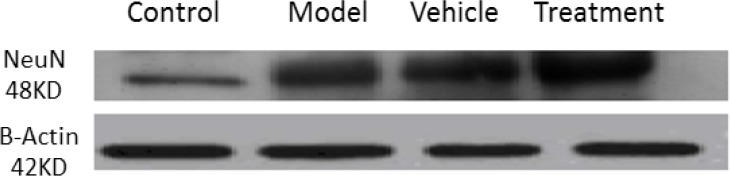
Western blot analysis of Neuronal-specific Nuclear (NeuN) protein expression. NeuN expression increased in the treatment group compared with the model, vehicle, and control groups ( ^*^ P<0.05; control, model, and vehicle groups vs. treatment group).

**Figure 10.**
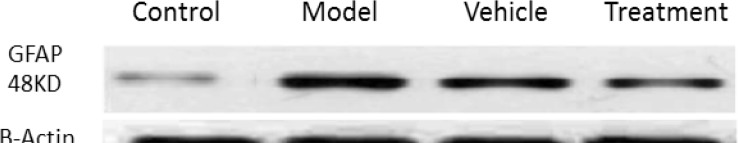
Western blot analysis of Glial Fibrillary Acidic Protein (GFAP) protein expressions

**Figure 11.**
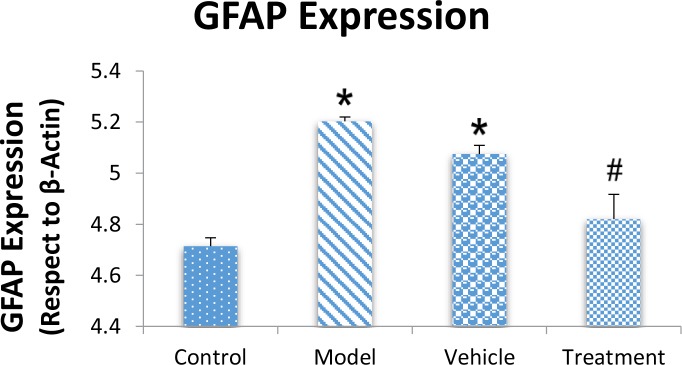
Western blot analysis of Glial Fibrillary Acidic Protein (GFAP) expression GFAP expression increased in the model and vehicle groups compared with the control group ( ^*^ P<0.05; model and vehicle groups vs. control group). And the BM-MSCs graft decreased the expression of the GFAP protein in the treatment group compared with the vehicle and model groups (#P<0.05; treatment group vs. model and vehicle groups).

We assessed the levels of GFAP and NeuN proteins in the rat hippocampus by Western blot analysis. The mean densities of the GFAP and NeuN bands were determined for all groups after normalizing against bands that corresponded with β-actin. According to Western blot analysis, NeuN protein expression increased in the treatment (0.608±0.009) group compared with the vehicle (0.45±0.024), model (0.44±0.012) and control (0.37±0.020) groups (P<0.05) (Figure 8 and Figure 9). TMT increased GFAP protein expression in the vehicle (5.07±0.03) and model (5.20±0.01) groups compared with the control (4.71±0.03) group (P<0.05) (Figure 10 and Figure 11). Cell graft decreased expression of the GFAP protein in the treatment (4.82±0.09) group compared with the vehicle and model groups (P<0.05) (Figure 10 and Figure 11).

## 4. Discussion

In this study, we observed the presence of damaged pyramidal cells in the CA1 region along with spatial memory deficit in rats injected with TMT.

TMT causes loss of the granular neurons of the dentate gyrus and the pyramidal cells of the CA1, activation of astrocytes, and increased expression of GFAP (Fiedorowicz et al., 2001; Geloso, Corvino, & Michetti, 2011; Geloso, Vinesi, & Michetti, 1998; Haga et al., 2002). After the injection of TMT, behavioral changes such as aggression and hyperactivity have been reported in animals (Geloso et al., 2011) along with impaired spatial and learning memory in the hippocampus (Earley et al., 1992; Walsh, Gallagher, Bostock, & Dyer, 1982). Our study results supported this finding. Following the injection of TMT, neural degeneration occurred similar to what is seen in neurodegenerative diseases such as Alzheimer (Jung et al., 2013).

Our findings showed that TMT administration caused an increase in traveled distance and escape latency of animals to reach the hidden platform. Our results supported the findings by Earley et al., who reported significantly damaged escape latency in rats that received TMT (Earley et al., 1992). TMT causes glutamate excitotoxicity, intracellular calcium overload, impairment of neurotransmission, and reduces acetylcholine and gluta-mate levels in the hippocampus. Ca2+ overload causes the generation of oxidative stress and subsequent neural degeneration. Disruption of Ca2+ homeostasis damages learning and memory function in animals (Florea, Dopp, & Büsselberg, 2005; Geloso et al., 2002; Ishikawa et al., 1997; Zhang, Li, Prabhakaran, Borowitz, & Isom, 2006).

According to the results, TMT treatment decreased the number of normal cells in the model and vehicle groups. Mignini et al., reported that TMT decreased neuronal viability and expression of the dopamine receptors and transporters in the hippocampus, and impaired spatial memory (Mignini et al., 2012). Geloso et al., have observed neuronal loss in the hippocampus and behavioral alterations in TMT-treated mice consistent with our findings (Geloso et al., 2002). TMT increases the number of lysosomes and big vacuoles, which indicate altered autophagy (Fabrizi et al., 2011). On the other hand, studies have shown that MSCs increase autolysosome formation. Autolysosomes are a modulator of autophagy that has been shown to enhance the elimination of Aβ in an Alzheimer disease model; thus, it is vital for neuronal homeostasis and survival (Shin et al., 2014).

According to the results, the treatment group that received BM-MSCs spent a shorter path and less time to reach the hidden platform during the training days. On the probe day, both distance and time spent increased in the target quarter, which indicated improved performance. Donega et al., reported that BMSCs improved cognitive function in a hypxia-ischemia modela (Donega et al., 2013). Yoo et al., reported that transplantation of MSCs increased functional recovery via both the proliferation of endogenous NSCs and the survival of newly generated neuroblasts in an ischemic brain (Yoo et al., 2008). Our findings showed that neuronal density decreased significantly in the CA1 hippocampus of the model group. On the other hand, after BM-MSCs transplantation, the number of normal neurons in the CA1 of the hippocampus increased in the treatment group compared with the model group. This increase could probably be due to the secretion of growth factors by BM-MSCs. Consistent with our study, Wu et al., reported that transplantation of BM-MSCs increased the number of cholinergic neurons and improved learning and memory in an Alzheimer disease rat model (Wu, Li, Feng, & Wang, 2007). Omprakash et al., observed human amniotic cell transplantation into the hippocampus of rats after injection of TMT restored the number of nerve cells in the CA1 and CA3 regions to near normal levels (Omprakash et al., 2011), which supported the results of the current study. Transplantation of BM-MSCs treat the damaged brain tissues by alleviation of the inflammatory response, secretion of neurotrophic factor, support of the endogenous recovery procedure, and migration to the injury region, with subsequent differentiation into neurons and astrocytes (Onteniente, 2013; Otero, Vaquero, Aguayo, Zurita, & Bonilla, 2011; Qin et al., 2013).

BM-MSCs secrete numerous neurotrophic and growth factors such as brain-derived neurotrophic factor, vascular endothelial growth factor, glial cell line-derived neurotrophic factor, and nerve growth factor that protect and induce regeneration of damaged tissue (Song, Mohamad, Gu, Wei, & Yu, 2013; Tanna & Sachan, 2014; L. Wei, Fraser, Lu, Hu, & Yu, 2012; Wei et al., 2013; Yang, Wu, Liu, & He, 2015). Brain-derived neurotrophic factor regulates neuronal survival during differentiation and regeneration of injured nerve cells. Neurotrophins have an essential role in neuroprotection and synaptic plasticity (Dimatelis, Russell, Stein, & Daniels, 2014; Lee et al., 2012). Intracerebral transplantation of MSCs improves neurological function. This may be due to the secretion of trophic factors and cytokines by the MSCs rather than their direct replacement of the damaged nervous tissue (Chen et al., 2001). These factors and the other chemokines secreted by MSCs can increase neurogenesis and activate adjacent astrocytes (Munoz et al., 2005). On the other hand, astrocytes can express several factors that increase neurogenesis (Nakayama, Momoki-Soga, & Inoue, 2003). These growth factors, along with cytokines secreted by BM-MSCs, activate mechanisms that can increase neurogenesis, angiogenesis and synaptogenesis (Donega et al., 2013; Li et al., 2008; Liu, Li, Zhang, Savant-Bhonsale, & Chopp, 2008). The proliferation of astrocytes increases after hippocampal damage. Astrocytes are a barrier that prevent the spread of damage (Li et al., 2008). After treatment with MSCs, the number of recently divided astrocytes decrease (Lee et al., 2010).

According to Western blot analysis, TMT increased GFAP protein expression in the vehicle and model groups compared with the control group. BM-MSCs transplantation decreased the expression of the GFAP protein in the treatment group compared with the vehicle and model groups. Transplantation of BM-MSCs increases the proliferation of NSCs that reside in the hippocampus. The new cells can also express neuronal and astrocyte markers (Munoz et al., 2005). In the present study, cell transplantation has led to a significant increase in the expression of NeuN protein in the treatment group compared with the vehicle and model groups. Based on the evidence, when BM-MSCs are transplanted into a neurodegenerative environment, they can differentiate into functional CNS neurons (Bae et al., 2007). Wu et al., have reported that a graft of BM-MSC in Alzheimer disease rat models improved learning and memory. Histological studies showed an increase in the number of neurons (Wu et al., 2007). This study, consistent with other studies, showed that cell transplantation might be useful in improving memory. Transplantation of MSCs could be a therapeutic potential for the treatment of behavioral defects (Chen et al., 2001).

## 5. Conclusion

The present study has shown that the transplantation of BM-MSCs increased the number of pyramidal neurons in the damaged hippocampus, improved behavioral performance, and reduced cognitive deficits induced by TMT administration. We suggest that neurotrophic factors might improve neurological function. However, further studies should investigate the effects of neurotrophic factors secreted by MSCs.

## Ethical Considerations

### Compliance with ethical guidelines

The Ethics Committee for the use of Laboratory Animals at Tehran University of Medical Sciences approved the present study.
